# Two-Spotted Ladybeetle *Adalia bipunctata* L. (Coleoptera: Coccinellidae): A Commercially Available Predator to Control Asian Citrus Psyllid *Diaphorina citri* (Hemiptera: Liviidae)

**DOI:** 10.1371/journal.pone.0162843

**Published:** 2016-09-15

**Authors:** Azhar A. Khan, Jawwad A. Qureshi, Muhammad Afzal, Philip A. Stansly

**Affiliations:** 1 University of Florida, Entomology and Nematology Department, Institute of Food and Agricultural Sciences, Southwest Florida Research and Education Center, Immokalee, Florida, United States of America; 2 University of Sargodha, Department of Entomology, Sargodha, Pakistan; Montana State University Bozeman, UNITED STATES

## Abstract

The Asian citrus psyllid *Diaphorina citri* Kuwayama (Hemiptera: Liviidae) is an economically important pest of citrus because it serves as a vector of the causal pathogens of huanglongbing (HLB) also known as citrus greening disease. The increased use of insecticides for control of *D*. *citri* negatively impacts several natural enemies including some effective ladybeetle species which are not available commercially. The two-spotted ladybeetle, *Adalia bipunctata* (Coleoptera: Coccinellidae) is found in some crop and forest ecosystems of Asia, Europe and North America and available commercially. It is known to attack aphids and mealybugs but there are no published records of feeding on psyllids. We evaluated suitability and preference of *A*. *bipunctata* for nymphs of *D*. *citri* compared to corn leaf aphid *Rhopalosiphum maidis* (Hemiptera: Aphididae) a global pest of cereal crops and prey for many predaceous insects. We also compared development and reproduction of *A*. *bipunctata* on these two species with frozen eggs of the Mediterranean flour moth *Ephestia kuehniella* (Lepidoptera: Pyralidae) at 25°C. Initially, more *D*. *citri* than *R*. *maidis* nymphs were consumed in the no-choice tests although final consumption by larva and adult of *A*. *bipunctata* did not differ in the choice and no-choice tests. Larval development was prolonged by one day on *D*. *citri* compared to *R*. *maidis* nymphs but did not differ between either of these diets and *E*. *kuehniella*. Larval survival to adult averaged 93–100% and was not impacted by diet. Adult life span did not differ between diets although those on *D*. *citri* and *R*. *maidis* nymphs weighed less and produced fewer but more fertile eggs than on *E*. *kuehniella* eggs. Significant reduction of *D*. *citri* nymphs averaging 54% was observed in colonies caged with adult *A*. *bipunctata* on field planted citrus. R° (net reproductive rate) was least for beetles fed *R*. *maidis*, but otherwise there were no significant differences in demographic parameters. Successful feeding, development and reproductive performance of *A*. *bipunctata* suggest its usefulness as biological control agent of *D*. *citri* as well as aphid species exemplified by *R*. *maidis*.

## Introduction

The Asian citrus psyllid [ACP] *Diaphorina citri* Kuwayama [Hemiptera: Liviidae] serves as a vector of the causal pathogens of huanglongbing [HLB] also known as citrus greening disease. Thus ACP is an economically important pest of citrus in HLB affected regions [1]. *Diaphorina citri* was first described in Taiwan [2], later found in Punjab Pakistan and the rest of the region [3]. *Diaphorina citri* was reported from Brazil in 1940 [4] and from Florida in 1998 [5]. Huanglongbing in Florida was detected in 2005 [6] one year after it was identified in Brazil in 2004 [7,8].

Biological control plays an important role in citrus pest management in Florida [9,10,11]. Ladybeetles, syrphid flies, lacewings and spiders are common predators of *D*. *citri*. Ladybeetles are considered major contributor to natural mortality of *D*. *citri* eggs and nymphs. Michaud and Olsen [12] found that *Olla v-nigrum* (Mulsant), *Harmonia axyridis* (Pallas), *Curinus coeruleus* (Mulsant), and *Exochomus childreni* (Mulsant) developed and reproduced on a diet of *D*. *citri* nymphs whereas *Cycloneda sanguinea* (L.) did not reproduce. Michaud [10] reported *H*. *axyridis*, *O*. *v-nigrum*, *C*. *sanguinea* and *E*. *childreni* as key predators of *D*. *citri* nymphs in central Florida. Qureshi and Stansly [11] attributed most of the 90–100% observed mortality of *D*. *citri* nymphs in southwest Florida citrus to ladybeetles *O*. *v-nigrum*, *C*. *coeruleus*, *H*. *axyridis* and *C*. *sanguinea*.

The use of insecticides has increased tremendously in Florida citrus with some growers using up to 12 sprays to control ACP and reduce incidence and intensity of HLB [13]. Such intense use of insecticides may accelerate selection for pest resistance and negatively impact naturally occurring biological control by ladybeetles, other predators and parasitoids thus reducing control of *D*. *citri* and promoting secondary pest outbreaks [14–17]. The populations of ladybeetles shown to be effective against *D*. *citri* have notably declined over recent years [Qureshi, unpublished data], presumably due to the increased use of insecticides. It is therefore important to evaluate the performance of commercially available natural enemies against *D*. *citri* and other pests in order to augment biological control. Commercially available predators that have been tested against *D*. *citri* include the convergent ladybeetle *Hippodamia convergens* Guérin-Méneville (Coleoptera: Coccinellidae), which developed and reproduced on the diets of *D*. *citri*, brown citrus aphid *Toxoptera citricida* Kirkaldy and green citrus aphid or spirea aphid *Aphis spiraecola* (Homoptera: Aphididae) [18]. Similarly a predatory mite *Amblyseius swirskii* Athias-Henriot (Acari: Phytoseiidae) was effective in reducing *D*. *citri* through feeding on its eggs and neonates [19].

The two-spotted ladybeetle *Adalia bipunctata* (L.) is a commercially available species used for aphid control in many countries [20–24]. It shares six percent of overall world market for aphid preferring predators and in Western Europe, is largely used for aphid control in the urban landscape (R. Timmer, Koppert BV, Netherlands). *Adalia bipunctata* was reported during a survey of predatory beetles in field crops and forests in Faisalabad, Pakistan [25]. Later, it was also reported among twelve species of predatory Coccinellids found in Chitral district of Pakistan [26]. However, its role against *D*. *citri* was never investigated. The objectives of our investigations were to evaluate the preference and suitability of larvae and adults of *A*. *bipunctata* for nymphs of *D*. *citri* compared to a common and available aphid, *Rhopalosiphum maidis* Fitch (Hemiptera: Aphididae) as well as survival, development and reproduction on these two species and frozen eggs of the Mediterranean flour moth *Ephestia kuehniella* (Lepidoptera: Pyralidae). We included *R*. *maidis* because it is one of the global pests of cereal crops, is commonly used in banker plants, and important to survival of several predators which forage multiple crops including *A*. *bipunctata*. *Ephestia kuehniella* eggs are commonly used in the laboratory to rear predators and support the development and reproduction of several ladybeetle species [12,18].

## Materials and Methods

### Study location, insects and experimental conditions

Experiments were conducted at the Southwest Florida Research and Education Center (SWFREC) of the University of Florida-IFAS, Immokalee, FL, USA (Latitude: 26.484 N, Longitude: 81.435 W). No permit or specific permission was required. These studies did not involve endangered or protected species. Colonies of *D*. *citri* and *R*. *maidis* were established at the SWFREC, Immokalee, FL. *Diaphorina citri* was reared on orange jasmine *Murraya paniculata* (L.), a close relative of citrus and one of the preferred hosts of *D*. *citri*. *Rhopalosiphum maidis* was reared on *Sorghum bicolor* (L.) Moench, a grass species cultivated for grain to feed animals and humans, and for ethanol production. Both colonies were maintained in a climate controlled glasshouse set at 28°C. *Adalia bipunctata* larvae were originally obtained from BIOBEST (BIOBEST-NV, USA Inc. Detroit MI 48201–2311) and reared through adulthood on frozen eggs of *E*. *kuehniella* (Koppert Biological Systems, Romulus MI 48174). Individual larvae were reared in experimental arenas (petri dishes 9 cm diameter by 1.5 cm high) and provided with water on a small cube of moist sponge. Upon emergence, adults were placed in 3-litre ventilated plastic jars (15 adults per jar) to initiate a colony which was maintained on eggs of *E*. *kuehniella*. Shoots of *M*. *paniculata* were provided as substrate for oviposition. The colony was maintained in an incubator (Percival, Model I36LLC8, Percival Scientific Inc. Perry, Iowa, USA) set to a photoperiod of 16:8 (L:D) at 25°C. Eggs and emerging larvae were collected on daily basis and kept under above conditions. Same conditions were used for the experiments.

### Choice and no-choice tests

First instar larvae or 4–5 day old adults of *A*. *bipunctata* were tested in choice and no-choice tests. Fifteen replicates for larva or 14 for adults were used for the three treatments; i) 20 nymphs of *D*. *citri* alone (no-choice test) ii) 20 nymphs of *R*. *maidis* alone (no-choice test) and iii) 10 nymphs each of *D*. *citri* and *R*. *maidis* together (choice test). Second and third instar nymphs of *D*. *citri* and *R*. *maidis* were provided to an individual larva or adult in experimental arena. The petri dish was covered with perforated parafilm to prevent escape of nymphs and to provide ventilation. Larvae and adults were starved for 24 hr prior to exposure to prey to motivate foraging [27]. Prey consumption was calculated at 3, 6 and 12 hrs after exposure by deducting the number of live nymphs from the total provided.

### Development and reproduction

Development of 48 h old larvae of *A*. *bipunctata* was evaluated through adult emergence followed by reproduction on three diets. Larvae were obtained from multiple egg batches of *A*. *bipunctata* stock colony and transferred individually using a soft camel hair brush to experimental arenas designated at random for one of the three diets; i) nymphs of *D*. *citri* ii) nymphs of *R*. *maidis* and iii) frozen eggs of *E*. *kuehniella*. *Diaphorina citri* nymphs on untreated citrus shoots, aphids on untreated sorghum leaves and *E*. *kuehniella* eggs on clean citrus leaves were provided to larvae on a daily basis through pupation. Water was provided on a small cube of sponge. Dates of larval death, pupation and adult emergence were recorded every 24-hrs. Freshly emerged adults were weighed on an A-200D balance (Denver Instrument, 5 Orville Dr. Bohemia, NY).

To determine effects of diet on larval instars of *A*. *bipunctata*, egg batches obtained from female *A*. *bipunctata* fed separately on diets of *D*. *citri*, *R*. *maidis* and *E*. *kuehniella* were kept in the incubator under conditions described above for the colony. Upon emergence, 1^st^ instar larvae of *A*. *bipunctata* were transferred to snap cap cups (Crystalware Clear Plastic Cups 9 oz) using camel hair brush and observed through pupation. There were 14 replicates for each diet. Rearing cup caps were perforated with needle for ventilation. Diets were changed every 24 hr and exuviae collected.

One week old adults of *A*. *bipunctata* developed on nymphs of *D*. *citri* or *R*. *maidis* and eggs of *E*. *kuehniella* were released in 5 liter plastic ventilated jars marked for respective diets and observed for mating. Ten mating pairs from each diet were transferred individually to experimental arenas and provided with the same diet on which they were reared. Psyllid nymphs on untreated *M*. *paniculata* shoots, aphids on untreated sorghum shoots and *E*. *kuehniella* eggs on a clean citrus leaf were provided daily *ad libitum*. A small 2 inch piece of folded paper towel and 3–4 clean leaves of *M*. *paniculata* were added as additional substrates for oviposition. Water was provided on a small cube of sponge. Eggs were counted and removed daily, along with the material on which they were laid and incubated in a separate Petri dish under the same conditions as above. Petri dishes with beetles were replaced every other day or earlier if eggs were laid on the dish. Newly hatched larvae were counted and removed every day with a soft camel’s-hair brush to avoid cannibalism of sibling or remaining eggs. Dates of beetle death were recorded.

### Field test of the *A*. *bipunctata* predation on *D*. *citri*

The field experiment was conducted in a citrus orchard at UF SWFREC, Immokalee, Florida. Twenty, 4 year-old 1.2–1.5 m tall ‘Hamlin’ orange trees were trimmed to encourage production of young shoots needed by *D*. *citri* to reproduce and develop. Twenty new shoots infested with *D*. *citri* nymphs were selected and examined to remove eggs and mature instars leaving a uniform cohort of 2^nd^ and 3^rd^ instars averaging 30 ± 9 per shoot. Selected shoots with nymphs were covered with sleeve cages to protect against additional oviposition, parasitism or predation. One week old adults of *A*. *bipunctata* from the laboratory colony fed on the eggs of *E*. *kuehniella* were released in 10 randomly selected sleeve cages at one adult per cage, leaving 10 caged shoots as a control without beetles. Cages were examined from 2–4 days to count psyllid adults and nymphs and transfer beetles to new shoots caged with counted numbers of 2^nd^ and 3^rd^ instar nymphs. Experiment was continued until all beetles died.

### Statistical analysis

A generalized linear model with Poisson errors (SAS PROC GENMOD) was used at *P* = 0.05 to evaluate treatment effects on number of *D*. *citri* or *R*. *maidis* nymphs consumed by larvae or adult *A*. *bipunctata* in choice and no-choice tests [28]. Data on larval survival until pupation or adult eclosion were analyzed by using the SAS GLIMMIXMACRO model with a logit link function to transform data [28]. Development times, adult weight, longevity, fecundity, and fertility were tested for diet effect using the Mixed procedure analysis and Tukey’s test for pairwise comparison of treatment means [*t*-tests] at a probability level of 0.05 [28]. Survival and fecundity data on all three diets given a sex ratio of 1:1 were used to estimate and analyze life table parameters of *A*. *bipunctata* according to the method proposed by Maia et al. [28, 29]. Percent reduction of psyllids in nymphal colonies with beetles was corrected using reduction in control colonies and Abbott’s formula [30] and analyzed using SAS Mixed model procedure. The relationship between female age and fecundity or colony size and consumption rate was evaluated with Pearson correlation statistics using the SAS Corr procedure [28].

## Results

### Choice and no-choice tests

Larvae or adult *A*. *bipunctata* did not show preference between nymphs of *D*. *citri* and *R*. *maidis* in choice tests (*P* > 0.05, Table 1). Although significantly more *D*. *citri* nymphs than *R*. *maidis* nymphs were consumed by larvae at 6 h after initiation of no-choice test (χ^2^ = 6.1, *df* = 1, *P* = 0.014, Table 1) no differences were seen at 12 h (*P* > 0.05). Adult consumption of *D*. *citri* nymphs was significantly more than *R*. *maidis* nymphs in the no-choice tests at 3 h (χ^2^ = 22.6, *df* = 1, *P* < 0.0001, Table 1) and 6 h (χ^2^ = 7.2, *df* = 1, *P* = 0.007, Table 1) but not at 12 h (*P* > 0.05). A total of 18–20 nymphs of one or both species were consumed within 12 h by a single larva or adult of *A*. *bipunctata* in choice and no-choice test.

**Table 1:** Mean number (±SEM) of nymphs of *D*. *citri* or *R*. *maidis* consumed by larvae and adults of *A*. *bipunctata* in two-way choice and no-choice tests.

	Choice test		No-choice test	
Observation time	*D*. *citri*	*R*. *maidis*	*D*. *citri*	*R*. *maidis*
Larva of *A*. *bipunctata*				
3 hours	3.1 ± 0.6 a	4.1 ± 0.6 a	4.4 ± 0.9 a	3.1 ± 1.0 a
6 hours	8.8 ± 0.3 a	9.1 ± 0.4 a	12.1 ± 1.5 a	9.1 ± 1.3 b
12 hours	9.3 ± 0.2 a	9.8 ± 0.1 a	19.4 ± 0.8 a	18.6 ± 0.2 a
Adult of *A*. *bipunctata*				
3 hours	5.1 ± 0.6 a	4.1 ± 0.5 a	9.9 ± 0.8 a	5.0 ± 0.7 b
6 hours	8.6 ± 0.4 a	7.3 ± 0.5 a	15.3 ± 0.9 a	11.6 ± 0.9 b
12 hours	9.7 ± 0.2 a	8.9 ± 0.5 a	19.6 ± 0.2 a	17.6 ± 0.7 a

Newly enclosed first instar larvae were used that were not exposed to any diet prior to the test. Adults from laboratory colony reared on eggs of *E*. *kuehniella* were starved for 24 h before the test. Means within a column followed by the same letter are not significantly different for the larva or adult (*P* > 0.05).

### Development and reproduction

**Fig 1:**
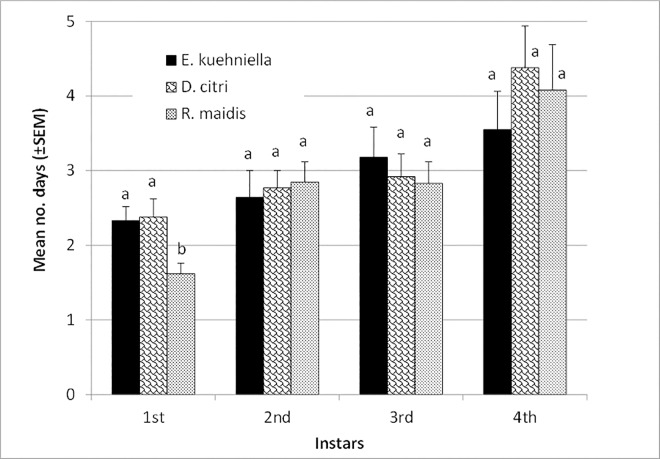
Mean (±SEM) development times of the larval instars of *A*. *bipunctata* on diets of *E*. *kuehniella* eggs, *D*. *citri* nymphs and *R*. *maidis* nymphs. Means with the same letter are not significantly different for respective instar (*P*>0.05)

No effect of diet on the egg incubation time was observed (*P* > 0.05, Table 2). Larval survival to pupation averaged 93–100% and adult emergence from pupae was 100% on all diets, without significant impact of diet on larval or pupal survival (*P* > 0.05). A significant effect of diet on larval development was only observed for the first of the 4 larval instars (F = 4.95; df = 2, 35; *P* = 0.0128, Fig 1). First instar larvae developed faster on *R*. *maidis* nymphs compared to *D*. *citri* nymphs (*P* = 0.0205) or *E*. *kuehniella* eggs (*P* = 0.0346), with no significant difference between these later two diets (*P* = 0.9814). These differences diminished in later instars (*P* > 0.05). Overall a significant effect of diet on the larval development was detected (F = 3.84; df = 2, 78; *P* = 0.0257, Table 2), which was slightly prolonged (*P* = 0.0271) on the nymphs of *D*. *citri* (11.8 ± 0.4 days) compared to *R*. *maidis* (10.7 ± 0.3 days). Development on *E*. *kuehniella* eggs (10.9 ± 0.3 days) and either of the two nymphal diets did not differ (*P* > 0.05, Table 2). Pupation time averaged 4.5–4.7 days with no significant diet effect (*P* > 0.05, Table 2). However, a significant effect of diet on the adult weight was seen (F = 4.88; df = 2, 40; *P* = 0.0127, Table 2), with heavier adults developing on *E*. *kuehniella* eggs compared to *R*. *maidis* nymphs (*P* = 0.0095) and those fed on *D*. *citri* nymphs being intermediate.

**Table 2:** Mean (±SEM) development times of immature stages and fresh weight of adult *A*. *bipunctata* reared on diets of *E*. *kuehniella* eggs, *D*. *citri* nymphs and *R*. *maidis* nymphs.

Diet	Egg (days)	Larva (days)	Pupa (days)	Adult weight (g)
*E*. *kuehniella*	3.6 ± 0.1 a	10.9 ± 0.3 ab	4.5 ± 0.2 a	0.009 ± 0.0004 a
*D*. *citri*	3.5 ± 0.1 a	11.8 ± 0.4 a	4.7 ± 0.4 a	0.008 ± 0.0003 ab
*R*. *maidis*	3.6 ± 0.1 a	10.7 ± 0.3 b	4.6 ± 0.4 a	0.007 ± 0.0004 b

Eggs and larvae were obtained from the adults developed on respective diets. Larval survival to pupation averaged 93–100% and 100% pupae resulted in adults on all three diets (*P* > 0.05). Means within a column followed by the same letter are not significantly different (P > 0.05)

Longevity of males and females was not impacted by diet (*P* > 0.05, Table 3). No diet-by-week interaction on fecundity was detected (F = 0.45; df = 10, 150; *P* = 0.9216), although significant effects of diet (F = 4.19; df = 2, 150; P = 0.0170) and date (F = 9.77; df = 5, 150; *P* < 0.0001) were seen.

**Table 3:** Mean (±SEM) adult longevity, life time fecundity and fertility of *A*. *bipunctata* on diets of *E*. *kuehniella* eggs, *D*. *citri* nymphs, and *R*. *maidis* nymphs.

Diet	Male longevity (days)	Female longevity (days)	Fecundity (no. of eggs/ female)	Fertility (% eggs hatched)	Fertility (no. of larvae/female)
*E*. *kuehniella*	34 ± 1.8 a	39 ± 3.2 a	300 ± 32.7 a	49 ± 3.5 b	103 ± 27.4 a
*D*. *citri*	32 ± 2.0 a	34 ± 1.9 a	222 ± 28.7 ab	66 ± 3.1 a	106 ± 25.1 a
*R*. *maidis*	30 ± 1.5 a	32 ± 2.1 a	186 ± 20.6 b	68 ± 3.6 a	86 ± 21.9 a

Means within columns sharing the same letter are not significantly different (P>0.05)

**Fig 2:**
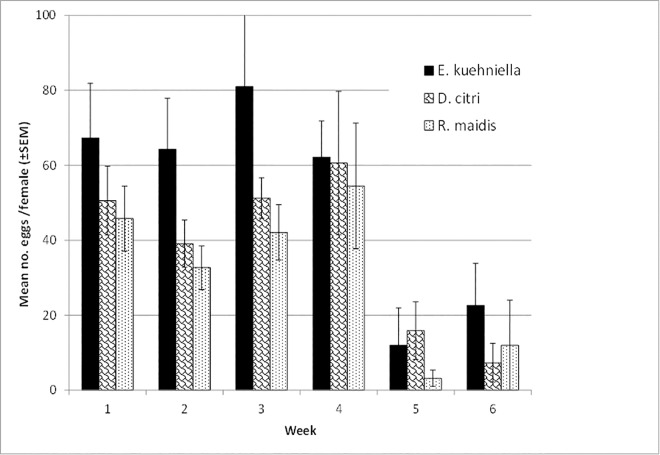
Mean (± SEM) weekly fecundity of *A*. *bipunctata* on diets of *E*. *kuehniella* eggs, *D*. *citri* nymphs, and *R*. *maidis* nymphs. Weekly means were not significantly different among diets (*P* >0.05).

In total, 441 oviposition events were observed from all females of which 44% occurred on leaves, followed by 29% on paper and 27% on petri dishes. Females fed on *R*. *maidis* nymphs produced significantly fewer eggs than those fed on *E*. *kuehniella* eggs (*P* = 0.0153), with no difference between diets of *R*. *maidis* and *D*. *citri* (*P* = 0.6943) or *D*. *citri* and *E*. *kuehniella* (*P* = 0.1163). A significant negative relationship between increasing female age and weekly fecundity rate was detected (*r* = -0.66, *P* = 0.003, Fig 2). Fertility of eggs was affected by diet (F = 9.2; df = 2, 275; *P* = 0.0001, Table 3) with only 49% hatching from females fed on *E*. *kuehniella* eggs compared to 66% and 68%, respectively, from the females fed on the nymphs of *D*. *citri* and *R*. *maidis* (*P* = 0.0008 and 0.0001, respectively) which did not differ (*P* = 0.6287) (Table 3). However, the number of live larvae per female was not significantly different among diets (F = 0.19; df = 2, 27; *P* = 0.8285, Table 3).

### Life table analysis

Net reproductive rate (*R*_*o*_) of *A*. *bipunctata* was greater on a diet of *E*. *kuehniella* eggs than on *R*. *maidis* nymphs (*t* = 3.16; *P* = 0.003) with the *D*. *citri* diet intermediate (*P* > 0.05, Table 4). No differences were seen among estimates of intrinsic rate of increase (*r*_*m*_), finite rate of increase (λ), generation time (*T*) or doubling time (*D*_*t*_) (*P* > 0.05) all indicating populations increasing on all three diets (Table 4).

**Table 4:** Means and 95% CL of the life table parameters *R*_*o*_ (net reproductive rate), *r*_*m*_ (intrinsic rate of increase), *λ* (finite rate of increase), *T* (generation time, days) and *Dt* (doubling time, days) of *A*. *bipunctata* on diets of *E*. *kuehniella* eggs, *D*. *citri* nymphs, and *R*. *maidis* nymphs.

Diet	*R*_*o*_	95% CL	*r*_*m*_	95% CL	*λ*	95% CL	*T*	95% CL	*Dt*	95% CL
*E*. *kuehniella*	150.2 a	113.24–187.15	0.37 a	0.28–0.45	1.44 a	1.32–1.56	13.61 a	10.76–16.46	1.88 a	1.44–2.32
*D*. *citri*	103.2 ab	73.00–133.37	0.33 a	0.29–0.37	1.39 a	1.34–1.45	13.96 a	12.00–15.91	2.08 a	1.84–2.32
*R*. *maidis*	86.3 b	64.56–107.95	0.33 a	0.26–0.40	1.39 a	1.29–1.49	13.41 a	10.59–16.23	2.08 a	1.66–2.50

Means within a column sharing the same letter are not significantly different (P > 0.05)

### *Adalia bipunctata* consumption of *D*. *citri* nymphs on citrus trees

**Fig 3:**
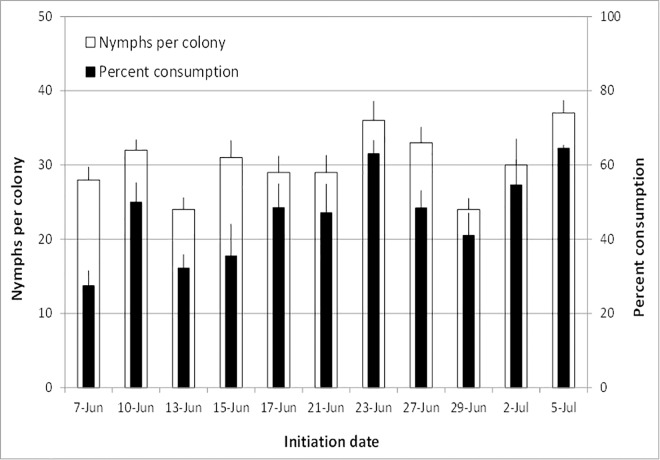
Mean (±SEM) number of *D*. *citri* nymphs in colonies developing on 4-year old ‘Hamlin’ orange trees, and percentage of nymphs consumed by *A*. *bipunctata* ladybeetles caged at one beetle per colony. Beetles were moved to new colonies every 2–4 days depending upon quantity of nymphs available to them. Percent consumption by beetles was corrected using Abbott’s formula (1925) to account for natural reduction in control colonies without beetles.

**Fig 4:**
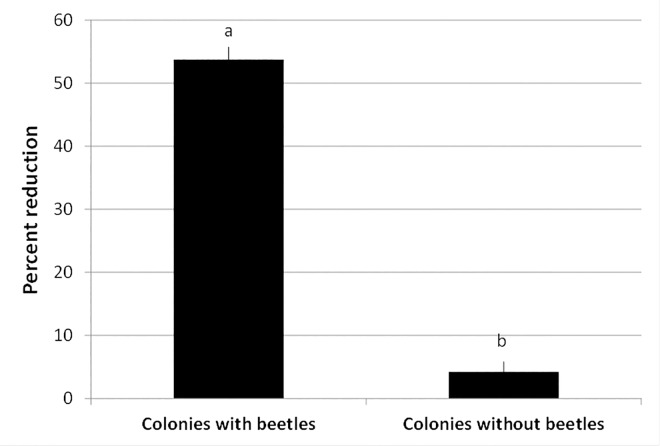
Total percentage reduction of nymphs (mean ± SEM) from all colonies of *D*. *citri* with and without *A*. *bipunctata* beetles. Reduction in colonies with beetles was corrected using Abbott’s formula (1925) to account for natural reduction in control colonies without beetles (*P*<0.05).

*Adalia bipunctata* beetles suppressed *D*. *citri* nymphs in all the tested colonies (*n* = 71, Fig 3). There was a significantly positive relationship between colony size, which ranged between 24–37 nymphs, and consumption rate (*r* = 0.77, *P* = 0.006, Fig 3). Overall, 54% reduction in nymphs was observed in colonies with beetles, which was significantly more than 4% in colonies without beetles (F = 379.69; df = 1, 18; *P* < 0.0001, Fig 4). On average, 136 ± 13 nymphs were consumed by each beetle.

## Discussion

Neither *A*. *bipunctata* larvae nor adults expressed preference between *D*. *citri* or *R*. *maidis* nymphs in choice tests. Apparently, cues of a visual, chemical or tactile nature involved in the orientation of *A*. *bipunctata* toward these prey did not influence selection. Initially, more *D*. *citri* than *R*. *maidis* were consumed in no-choice tests, although 90% or more of the 20 nymphs offered were consumed after 12 h regardless of prey species. These findings suggest that both *D*. *citri* and *R*. *maidis* were equally preferred and suitable as food for *A*. *bipunctata*.

The apparent suitability of *D*. *citri* and *R*. *maidis* for *A*. *bipunctata* translated into successful larval survival to adulthood averaging 93% on either prey species, and not significantly different from the 100% survival on eggs of *E*. *kuehniella*. However, larval development on nymphs of *D*. *citri* was prolonged by one day compared to *R*. *maidis* nymphs though not compared to *Ephestia* eggs. Late instar *D*. *citri* nymphs have well developed wing buds that may serve as deterrence as well as reduce consumable body contents compared to *R*. *maidis* nymphs or *Ephestia* eggs which can be completely consumed. Beetle larvae may have spent more time and energy in handing *D*. *citri* nymphs containing mixed population of young and mature instars compared to *R*. *maidis* nymphs or *Ephestia* eggs, resulting in delayed development. However, suitability of young instar *D*. *citri* nymphs compared to *R*. *maidis* nymphs was not compromised in the choice or no-choice tests where both species were consumed at the same rate over 12 h. These were young instars with undeveloped wing buds and thus probably easy for the larvae to handle and consume. Although larvae on a diet of *D*. *citri* nymphs took somewhat longer, to develop, they were able to meet their nutritional requirements as indicated by adults that were not different in weight from those developed on *R*. *maidis* nymphs or *Ephestia* eggs. However, adults developed on *R*. *maidis* nymphs weighed less than those from *Ephestia* eggs. In addition to some possible nutritional advantage, eggs are easy to handle by the predators compared to nymphs which are mobile and provide some resistance when attacked.

Females lived longer than males irrespective of diet although diet did not affect longevity of either gender. Fecundity decreased with female age, and was greatest during first four weeks on all three diets. Reproductive performance on the two nymphal diets was comparable and better compared to *Ephestia* eggs except reduced fecundity on *R*. *maidis*. De Clercq et al. [23] also observed improved fertility of *A*. *bipunctata* on an aphid diet compare to *Ephestia* eggs. Reproductive performance of *H*. *convergens* on nymphs of *D*. *citri*, *T*. *citricida and A*. *spiraecola* was comparable and better compared to *Ephestia* eggs [18]. However, fecundity and fertility of the *C*. *coeruleus*, *E*. *childreni*, *H*. *axyridis*, and *O*. *v-nigrum* fed with frozen *D*. *citri* nymphs and *Ephestia* eggs did not differ [12] indicating that freezing could have reduced the nutritional value of the nymphs.

All life table parameters predicted increasing populations of *A*. *bipunctata* on all three diets without significant differences except *R*_*o*_ which was reduced on the *R*. *maidis* nymphs compare to *Ephestia* eggs. Estimated intrinsic rate of increase was higher and generation time shorter in the present study than reported for *A*. *bipunctata* on the green peach aphid *Myzus persicae* (Sulzer) [31] or *H*. *convergens* on the nymphs of *D*. *citri*, A. *spiraecola*, *T*. *citricida* or *Ephestia* eggs [18]. The generation time of *A*. *bipunctata* observed in the present study was also much shorter than that of *O*. *v-nigrum*, *H*. *axyridis*, *E*. *childreni*, *C*. *sanguinea* and *C*. *coeruleus* [12]. These results indicate that *A*. *bipunctata* is another important candidate predator of *D*. *citri* as well as of *R*. *maidis*. The addition of commercially reared predators may counteract the pesticide-caused reductions of natural enemies in the orchard environment.
